# Functionalized hydrogels in neural injury repairing

**DOI:** 10.3389/fnins.2023.1199299

**Published:** 2023-06-19

**Authors:** Wenqian Zhao, Hui Tu, Jianxiao Chen, Jing Wang, Haoting Liu, Fengshou Zhang, Jing Li

**Affiliations:** ^1^College of Medical Technology and Engineering, Henan University of Science and Technology, Luoyang, China; ^2^Department of Nephrology, The Fourth Affiliated Hospital, Zhejiang University School of Medicine, Yiwu, Zhejiang, China; ^3^Office of Science and Technology, Henan University of Science and Technology, Luoyang, China

**Keywords:** functionalized hydrogel, nerve injury, tissue engineering, nerve regeneration, nanomaterial

## Abstract

Repairing injuries to the nervous system has always been a prominent topic in clinical research. Direct suturing and nerve displacement surgery are the primary treatment options, but they may not be suitable for long nerve injuries and may require sacrificing the functionality of other autologous nerves. With the emergence of tissue engineering, hydrogel materials have been identified as a promising technology with clinical translation potential for repairing nervous system injuries due to their excellent biocompatibility and ability to release or deliver functional ions. By controlling their composition and structure, hydrogels can be Functionalized and almost fully matched with nerve tissue and even simulate nerve conduction function and mechanical properties. Thus, they are suitable for repairing injuries to both the central and peripheral nervous systems. This article provides a review of recent research progress in functionalized hydrogels for nerve injury repair, highlighting the design differences among various materials and future research directions. We strongly believe that the development of functionalized hydrogels has great potential for improving the clinical treatment of nerve injuries.

## Introduction

The nervous system is a crucial component of living organisms, composed of the brain, spinal cord, and peripheral nervous tissue, and consists of abundant neurons and supportive glial cells that form neural circuits, regulating life activities and transmitting physiological information (Brodal, 2004; Sanes et al., 2011). In general, nerve damage or injury may result in the death of endogenous nerve cells at the lesion site, making spontaneous regeneration challenging and leading to abnormal or deteriorating organ functions, and even patient death. For instance, spinal cord fractures or dislocations due to accidents such as trauma, traffic accidents, falls, or sports injuries can cause nerve damage, leading to gradual deterioration of organ functions and consciousness below the injury site, and even paralysis in severe cases. Moreover, the high morbidity and mortality rates of nerve damage make it a significant public health issue worldwide, causing enormous psychological pain and economic burden to patients and their families (Karsy et al., 2019). Therefore, nerve injury repair is a topic of great interest, and the induction of nerve cell regeneration in the damaged area to restore the neural circuit and recover patients’ motor function is currently a crucial issue in the field of basic medical research and clinical transformation practice related to nerve damage treatment.

In the meantime, recent advances in tissue engineering and regenerative medicine have highlighted three crucial factors that play a key role in reshaping neural tissue structure and function. These include seed cells, biomaterial scaffolds, and bioactive factors (Schmidt and Leach, 2003; Gu et al., 2014). Usually, neural stem cells are used as seed cells for neural repair, and biomaterial scaffolds act as carriers for both seed cells and bioactive factors during stem cell transplantation. Other than that, an ideal biomaterial scaffold should provide anchoring sites for stem cells to adhere and grow, as well as induce their proliferation and differentiation within the microenvironment, ultimately leading to the formation of functional neural tissue with mechanical stability.

**Figure 1:**
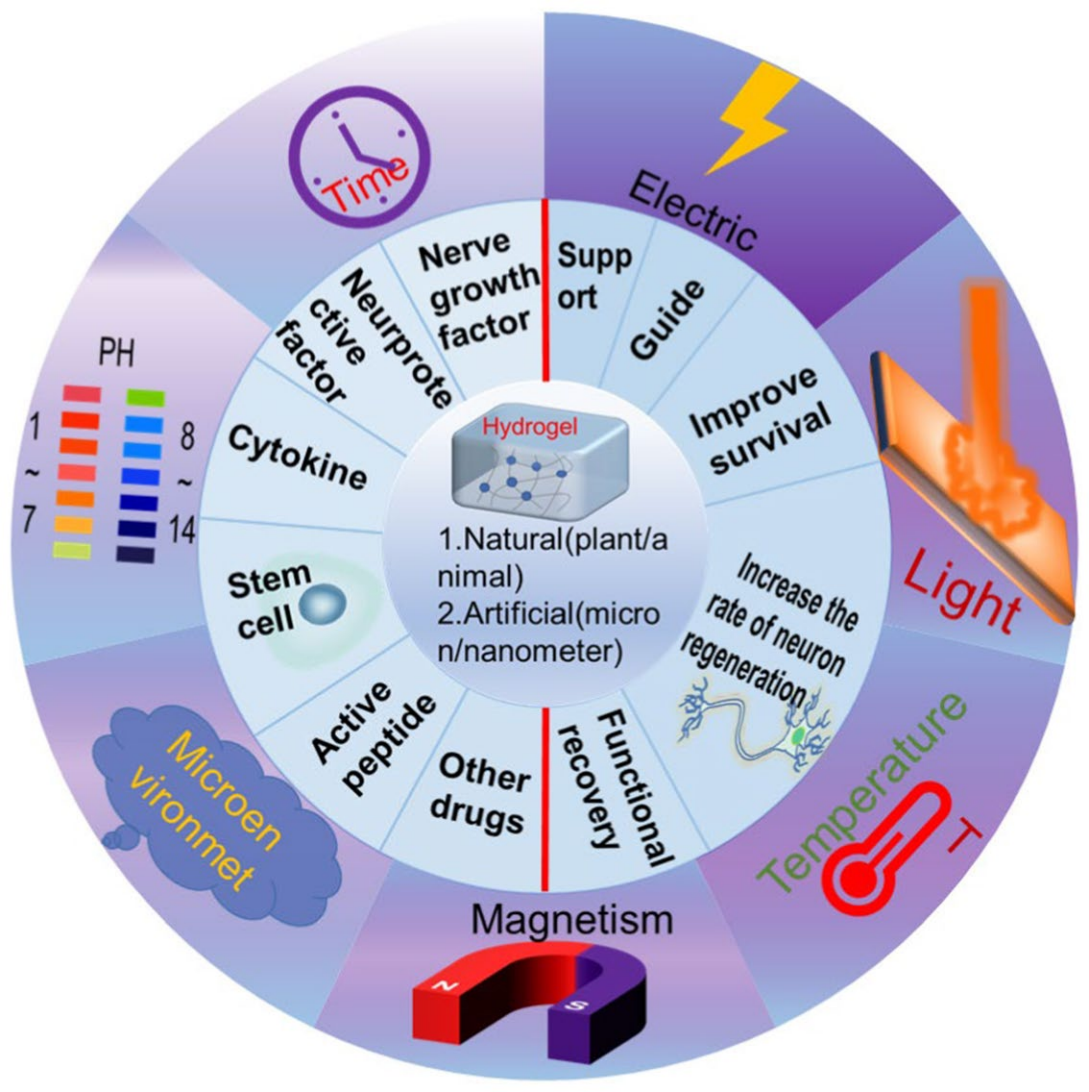
Schematic illustration showing the classification of functionalized hydrogels and remote control to realize the various functions.

As a scaffold material widely used in neural tissue engineering, hydrogel has the following properties. Hydrogels are highly porous network materials that result from crosslinking hydrophilic polymers through both physical and chemical processes (Ullah et al., 2015). They possess excellent biocompatibility, and as such, are widely used in the diagnosis and treatment of various diseases (Hoffman, 2012; Caló and Khutoryanskiy, 2015). The physicochemical and structural microenvironment of hydrogels resembles that of the extracellular matrix of neural tissue, and their viscoelastic property is highly compatible with biological tissue. Moreover, hydrogels provide attachment sites and a three-dimensional space for the growth, migration, proliferation, and differentiation of transplanted neural cells. Additionally, hydrogels exhibit tissue affinity on their soft and wet surfaces, making them ideal for *in vivo* cell scaffolding (Nisbet et al., 2008). To clarify, this mini review is structured around various strategies for distinct hydrogel material designs; however, it is important to note that due to differences in cellular microenvironments, cell types, and post-injury repair capabilities between the central and peripheral nervous systems (CNS and PNS, respectively), the responses to biomaterials vary. Consequently, the requirements for material design diverge as well. For instance, when designing biomaterials, the degradability of the material is a crucial factor. Biomaterials designed for CNS repair must degrade within an appropriate time frame to prevent long-term disruption to surrounding tissues, while in PNS repair, the degradation rate of biomaterials should correspond with the rate of nerve regeneration to ensure adequate support for nerve growth. Another example is the variation in biomaterial types for CNS and PNS injuries. CNS injury repair materials may encompass scaffolds, gels, nanoparticles, and other components with the primary aim of providing physical support, promoting cell growth and differentiation, and releasing growth factors. In contrast, PNS injury repair materials typically involve nerve conduits, biofilms, and the like, with the main functions of guiding nerve growth and supplying extracellular matrix support. Furthermore, drug delivery to the CNS can be challenging due to the blood–brain barrier. As a result, when designing biomaterials for CNS repair, it is worth considering the development of materials with drug carrier capabilities to enhance the efficiency of drug delivery to the damaged site. In PNS repair, however, the limitation imposed by the blood–brain barrier is less significant. In summary, the design of biomaterials for CNS and PNS injury repair necessitates a comprehensive understanding of their distinct biological, biomechanical, and biocompatibility aspects. Thus, this article reviews the fundamental research in neural injury repair that utilizes various functionalized hydrogels, explores their potential for clinical practice, and discusses the synthesis strategies and application conditions of different types of hydrogels (Figure 1).

## The supporting role: fundamental and key function of hydrogels in repairing nerve injuries

Hydrogels play a crucial role in nerve injury repair by providing physical support, enhancing mechanical and guiding capabilities, and promoting nerve regeneration and repair. The polymer chains within hydrogels can be crosslinked to form a three-dimensional network that enhances their mechanical properties, such as toughness, strength, and elasticity, allowing them to closely mimic physiological tissue structures (Lesný et al., 2002; Ma et al., 2022). Furthermore, the strength and elasticity of hydrogels can be adjusted by controlling their crosslinking density and structure, and subsequent adsorption of water molecules can increase their toughness and durability. The three-dimensional network structure formed by hydrogels at the microscale provides physical support to the surrounding tissues and cells, promoting oriented cell growth, facilitating the generation of new neural tissue and blood vessels, and enhancing the supply of oxygen and nutrients to support neural cell growth and regeneration (Deligkaris et al., 2010; Xia and Chen, 2022). Moreover, hydrogels can effectively fill damaged areas of neural tissue, reducing tissue gaps, forming supportive structures, and aiding in neural tissue repair (Štulík and Syková, 2008). Hydrogels can also be used as a carrier for bioactive molecules, making them a promising biomaterial for promoting neural tissue regeneration and repair (Ma et al., 2022).

Overall, the supportive role of hydrogels in neural injury repair is based on their formation of a three-dimensional network with uniform micro-pores, excellent biocompatibility, and the ability to promote neural regeneration and repair. The combination of these advantageous properties makes hydrogels a crucial and effective material for nerve repair, resulting in the survival and functional recovery of neurons, and providing hope for the millions of people affected by nerve injuries.

## Application of various functionalized hydrogels in neural injury repair

### Dopamine-functionalized hydrogels

Dopamine, a neurotransmitter, participates in numerous physiological and pathological processes of the CNS, including emotion, behavior, memory, attention, motivation, and reward (Wise, 2004; Iversen and Iversen, 2007). Furthermore, dopamine plays a crucial role in regulating the morphology, growth, and migration of neurons to the site of injury, which impacts the formation and connection of neurons and contributes to the growth, development, and repair of neurons (He et al., 2022; Lindholm and Saarma, 2022; Silva et al., 2022). Recent studies have revealed that dopamine also facilitates the growth and development of neurons, promotes synaptic formation and connection, and encourages the differentiation of neurons, allowing them to assume different roles in the nervous system (Kim et al., 2002; Chinta and Andersen, 2005). Therefore, incorporating dopamine into hydrogels can potentially enhance the ability of hydrogels to promote neural regeneration and repair.

The neural regulatory function of dopamine makes it a promising candidate for combining with hydrogels for nerve injury repair. Studies have shown that the failure of axonal regeneration in the CNS is closely related to the formation of glial scar after injury (Chen and Zhu 2016). However, dopamine-functionalized hydrogels have been found to be effective in promoting the differentiation of neural stem cells (NSCs) and the growth of synapses, inhibiting the formation of glial scars following spinal cord injury, and facilitating axon regeneration (Zhou, 2018).

Furthermore, regarding the optimization of dopamine binding to hydrogels, several studies have shown that it can be achieved through chemical reactions (Carballo-Molina et al., 2016; Adil et al., 2017), or by physisorption (Yang et al., 2012; Pei et al., 2020). The most common approach for dopamine-functionalized hydrogels is the oxidative polymerization reaction of dopamine under alkaline conditions, which generates a polymer compound called polydopamine (Tang et al., 2019; Chen et al., 2020). Polydopamine can then covalently bond with numerous hydrogel materials, such as dopamine-functionalized poly (lactic-co-glycolic acid) (PLGA) hydrogel. Another approach to combine dopamine with hydrogels is physical adsorption, which depends on the interaction between the aromatic ring structure of dopamine molecules and the aromatic functional groups on the hydrogel surface (Kim et al., 2014; Soylu et al., 2021). For instance, dopamine can be physically adsorbed onto gelatin hydrogels to impart them with dopamine-functionalized characteristics. A thorough comprehension of the chemical and physical interactions between dopamine and hydrogels is crucial in developing dopamine-functionalized hydrogels for neural repair applications.

Dopamine-functionalized hydrogels have shown promising potential in promoting neural stem cell adhesion and proliferation, with the ability to modulate the proliferation of neural stem cells through dopamine content adjustments. Moreover, these hydrogels have demonstrated an increase in the rate of neuronal differentiation, synapse formation promotion, and neural network development facilitation. Additionally, dopamine-functionalized hydrogels have been found to improve the survival rate of neural stem cells and enhance the regenerative capacity of neural tissue by modulating the extracellular environment of neural stem cells. As shown in Table 1, more and more studies have shown that the combination of dopamine and hydrogel is effective in repairing nerve injury.

**Table 1:** Representative studies of dopamine-functionalized different hydrogels and their biological properties in nerve injury repair.

Hydrogel synthetic materials	Nerve damage	Types of study	Effects on biological performances	Ref.
A photothermal responsive cell-laden self-rolling poly-N-isopropylacrylamide (PNIPAM) hydrogel	PNS	*In vitro*	Promote the growth of Schwann cells	Li et al. (2019)
Gelatin methacrylate (GelMA)	CNS	*In vitro*	Supporting stem cell growth and improving neural differentiation of NSCs	Li et al. (2019)
Injectable hydrogels	CNS	*In vivo*	Have a positive repair effect on spinal cord injury	Li et al. (2019)
Gelatin methacryloyl (GelMA) hydrogel	CNS/PNS	*In vivo*	Promoting the differentiation of mesenchymal stem cells into neuron-like cells	Li et al. (2019)

### Conductive hydrogels

The nervous system primarily communicates through electrical signals, which are critical in the development, maturation, and regeneration of nervous tissue. Inter-cellular signal transmission predominantly occurs *via* the extracellular matrix, and the integration of conductive substrates into the cellular microenvironment promotes inter-cellular electrical signal transmission. Therefore, maintaining the function of neurons heavily relies on the stimulation and transmission of electrical signals. Peripheral nerve injury can result in neurologic disorders, chronic pain, paralysis, and even disability by disrupting the electrical signal transmission between the brain and the body. Autologous nerve transplantation is commonly used to repair peripheral nerves; however, this method is affected by various factors such as a shortage of donors, long-term excessive tension, synaptic regeneration disorder, and severe nerve interruption that is difficult to suture. Conductive hydrogels have become a preferred alternative to autologous nerve transplantation due to their biocompatibility and the advantages of conductive polymers that can transmit electrical signals within nerve tissue. Additionally, conductive hydrogels have been found to effectively use electrical signals to promote neural tissue regeneration (Dong et al., 2020).

Extensive research has been conducted on biocompatible and conductive biomaterials that promote neural tissue regeneration (Guarino et al., 2013; Uz and Mallapragada, 2019; Park et al., 2020). Conductive hydrogels are a specific type of hydrogel material with electrical conductivity, containing conductive substances such as conductive polymers (polypyrrole, polyaniline, and polystyrene), metallic elements (silver, copper, gold, and iron), carbon materials (graphene, carbon nanotubes), and metal oxides (indium tin oxide, and aluminum oxide), as compared to traditional hydrogel materials (Wang et al., 2018; Zhou et al., 2022). These conductive substances can be combined with hydrogels through direct mixing, chemical reduction, or electrochemical deposition.

Conductive hydrogels find application in electronic devices, biosensors, and smart medical fields (Xu et al., 2020). However, it is worth noting that the conductive properties of these materials can vary greatly. For example, while metals display excellent conductivity, their biocompatibility is relatively poor. On the other hand, carbon-based materials can enhance the mechanical strength of hydrogels, but achieving homogeneous dispersion is challenging, which subsequently affects the conductivity performance. Conductive hydrogels have the potential to establish a neural-electronic interface that connects electrons and nerve cells, enabling electronic and ionic transport to simulate electrical signal transmission between nerve cells. Research has demonstrated that the conductive properties of conductive hydrogels can create an electrical stimulation environment that promotes the growth and regeneration of neural cells (Liu et al., 2017; Park et al., 2020; Cai et al., 2022). For example, conductive hydrogel conduits with a gradient of growth factors can promote the regeneration of peripheral nerves and muscle fibers in mice, holding significant potential for repairing peripheral nerve injuries (PNIs) and muscle atrophy in diabetic patients (Liu et al., 2017; Park et al., 2020; Cai et al., 2022). Furthermore, using conductive polymer hydrogels to repair spinal cord injuries in mice has resulted in the regeneration of spinal cord neurons and the restoration of limb motor function (Zhou et al., 2018; Guo et al., 2019).

Conductive hydrogels, specifically poly (acrylic acid)/polypyrrole (PAA/PPy) hydrogels, have been successfully utilized as scaffold materials for facilitating the differentiation of neural stem cells into neurons under electrical stimulation, as reported by (Milani et al., 2022). Additionally, the combination of these hydrogels and electrodes can be employed for neural electrical stimulation, modulation, and recording of neural electrical activity signals in the cerebral cortex, as mentioned in (Khan et al., 2022). Conductive hydrogels not only possess suitable physicochemical properties for cell growth but also exhibit electrical conductivity, which enables them to provide additional electrical stimulation to nerve cells. This capability can support the restoration of interrupted conduction pathways and maintain the endogenous electrical microenvironment for nerve regeneration. Therefore, the interaction between conductive hydrogels and nerve cells is a critical area of research in the field of neural repair and is considered one of the popular technologies for achieving nerve regeneration and repair. As shown in Table 2, more and more studies have shown the effectiveness of conductive hydrogels for repairing nerve injury.

**Table 2:** Representative study of conductive hydrogels and their biological properties in nerve injury repair.

Hydrogel synthetic materials	Nerve damage	Types of study	Effects on biological performances	Ref.
Conductive hydrogel catheter with growth factor gradient	PNS	*In vivo*	Promote repairing of peripheral nerve injury and inhibit the atrophy of muscles for diabetics	Li et al. (2019)
Exosome-loaded conductive hydrogel	CNS	*In vivo*	Promoting neural stem cell differentiation and axon regeneration	Li et al. (2019)
Injectable and biodegradable conductive hydrogels	CNS	*In vitro*	Promote the differentiation of neural stem cells into neurons, inhibit the formation of glial cells and scars	Li et al. (2019)
Hybrid electrically conductive hydrogels	PNS	*In vivo*	Promoting myelin regeneration of injured axons	Li et al. (2019)

However, conductive materials present various challenges in their application within the field of biomedicine. Firstly, they may be difficult to process and degrade, with poor uniform dispersion of carbon-based materials. Surface modification and controlling polymerization conditions have been suggested as potential solutions to these issues. Secondly, further study is needed to fully understand the synergistic mechanism between electrical stimulation and conductive materials, to better meet the challenges of long-distance nerve injury repair. Finally, the long-term cytotoxicity, biocompatibility, and metabolism of conductive materials within the human body remain areas of active research focus (Khan et al., 2022).

## Extracellular vesicles functionalized hydrogels

The nervous system communicates mainly through electrical signals, and in the CNS, neurons are responsible for signal transduction. The severe inflammatory reaction after nerve injury can lead to the death of neurons and the formation of glial scar, which affects the repair of CNS injury. Therefore, ideal CNS repair materials need to have properties that minimize the occurrence of inflammatory reactions (Raposo and Stahl, 2019). Studies have shown that extracellular vesicles (EVs) contain a diverse array of biological molecules, such as cell factors, proteins, and nucleic acids, and have multiple biological functions, including promoting cell proliferation and reducing inflammation (Raposo and Stahl, 2019). Previous studies have indicated that EVs derived from cells possess robust regenerative and reparative capabilities in various systems, including the musculoskeletal (Alcaraz et al., 2019), cardiovascular (Adamiak et al., 2018), liver injury (Psaraki et al., 2022), kidney injury (Aghajani Nargesi et al., 2017), traumatic brain injury (Mondello et al., 2018) and others. These vesicles have significant potential as a “cell-free” therapeutic approach for regenerative medicine and may serve as a replacement for stem cells. Notably, many cells in the peripheral nervous system, such as small glial cells and astrocytes, are capable of secreting different types of EVs, particularly following nerve injury.

And EVs can participate in protein synthesis in neurons, promote axonal regeneration, and inhibit axonal degeneration through various mechanisms. One such mechanism involves the binding of vesicular membrane proteins with target cell membrane proteins through ligand-receptor interactions (Liu et al., 2021). Another mechanism is that vesicular proteins can activate signaling pathway proteins on the surface of target cells after vesicle degradation, leading to a series of biological responses (Guo et al., 2021; Lee et al., 2022). Loading functionalized hydrogels with EVs is a novel therapeutic approach that can promote neural repair through various pathways such as enhancing the proliferation and migration of neural cells, improving neuronal survival, and restoring function. Studies have shown that EVs derived from skin-derived precursor Schwann cells (SKP-SC-EVs) accelerate the recovery of motor, sensory and electrophysiological functions in rats, promote the growth of regenerative axons and the formation of myelin sheaths, reduce the atrophy of target muscles caused by denervation, and promote the neurite growth of motor neurons (MNs) and sensory neurons, which is helpful for PNI repair (Yu et al., 2021; Jiang et al., 2022; Zhang et al., 2022). This promising approach may hold significant potential for the development of regenerative medicine therapies for neural injury and degenerative disorders.

Various studies have demonstrated the potential of stem cell-derived extracellular vesicles (EVs) and exosomes to reduce inflammation, inhibit cell apoptosis, and mitigate the impact of neural damage on surrounding tissues. For instance, researchers have loaded human mesenchymal stem cell-derived EVs onto chitosan-based functional hydrogels to repair peripheral nerve injury in rats. The results of the study indicated that the EV-loaded functional hydrogels promoted neuronal cells proliferation and migration, while reducing inflammation and cell apoptosis, leading to a significant improvement in neural damage repair (Ju et al., 2023). Another study utilized exosomes derived from mouse neural stem cells loaded onto poly (ε-caprolactone) based functional hydrogels and reported a significant increase in the survival and regeneration of spinal cord neurons, accompanied by a reduction in inflammation and glial scar formation (Xie et al., 2022). Moreover, there have been studies loading exosomes containing miRNA into collagen hydrogels for neural injury repair, demonstrating that they not only promote neuronal growth and regeneration but also improve the structure and function of damaged neural tissue (Lei et al., 2019; Xu et al., 2021; Zhang et al., 2021). In this study, the 3D fiber-hydrogel scaffold delivered axon microRNA (miR) to the injured site and repaired it in a non-viral manner. In the presence of methylprednisolone, the role of axon miRs in promoting mature axon regeneration is not affected, which can reduce the inflammatory response. More importantly, axon miRs in the presence of methylprednisolone reduced cyst formation and provided a tendency to improve functional recovery (Zhang et al., 2021).

Despite the promising therapeutic effects of EVs in regenerative medicine research, such as tissue repair and regeneration, their naturally low quantity and difficulty in controlling them present challenges in terms of extraction efficiency and purification yield, which has limited their research and application (Ingato et al., 2016; Usman et al., 2018). Therefore, it is crucial to improve the quality and yield of EVs, which will be a key focus for future research. Furthermore, before they can be effectively applied in clinical settings, more studies are needed to validate their safety and efficacy. But with the progress of technology, we believe that these issues will be addressed soon, and look forward to further development in this field.

## Nanomaterials functionalized hydrogel

In recent years, research on nano-functionalized hydrogels for neural injury repair has gained significant attention due to the expanding and enhancing of hydrogel functionality by nanomaterials (Rafieian et al., 2019; Kailasa et al., 2022). Nanomaterials can provide more growth factors and cell adhesion molecules, promoting the regeneration and growth of neural cells, while hydrogels provide a supportive and protective environment that facilitates the directional growth of neural cells. Additionally, nanomaterials can serve as drug carriers, enhancing drug loading and release efficiency for neural repair drugs, and even enabling intelligent and precise controlled release. As a result, nanomaterial-based hydrogels offer promising potential for the development of effective neural injury repair strategies. Hydrogels can limit drug diffusion and degradation, thus increasing drug concentration and duration at the treatment site. Furthermore, nanomaterials can enhance the mechanical properties and stability of hydrogels, resulting in improved durability and lifespan of the material (Li and Mooney, 2016). With their large and specific surface area, nanoparticles can increase drug delivery efficiency, and in combination with hydrogels, the use of nanomaterials can minimize immune reactions. Composites of nanoparticle-metal-hydrogel have been found to promote neuronal growth and have the potential to facilitate nerve regeneration and repair.

Nanoparticle biosensors have been combined with hydrogels to promote nerve regeneration and repair by enhancing neuronal proliferation and differentiation. Nanocarbon tube hydrogels have shown promise in nerve regeneration by promoting neuronal proliferation, reducing inflammation, and increasing the rate of neuronal regeneration (Ye et al., 2021). Similarly, nanoparticle proton pump hydrogels have been developed to reduce inflammation during nerve regeneration and promote neuronal regeneration, improving the effectiveness of nerve injury repair (Mendiratta et al., 2019). Graphene oxide nanosheets have also been incorporated into gelatin hydrogels to create a composite material for nerve regeneration, which has been shown to promote the proliferation and differentiation of neural stem cells and achieve nerve regeneration *in vivo*. The incorporation of micropatterns and bioactive substances into the inner wall of nerve-guide conduits (NGCs) can effectively regulate the behavior of Schwann cells, axon elongation, and macrophage phenotype, ultimately promoting the regeneration of injured nerves. In a recent study, 3/3, 5/5/, 10/10, and 30/30 μm linear micro-ribbons were prepared on poly (D, l-lactic acid-co-caprolactone) (PLCL) films. Surface ammonolysis and electrostatic adsorption of graphene oxide (GO) nanosheets were performed. This material has demonstrated great potential for promoting nerve regeneration (Zhang et al., 2020). Additionally, magnetic nanoparticle (MNPs) and gelatin hydrogel composites have been developed, which exhibit a significant magnetic guiding effect, facilitating the directional differentiation of neural stem cells and promoting nerve regeneration (Pavón et al., 2019). Curcumin-loaded mesoporous silica nanoparticles (MSN-CCM) dispersed in hydrogels have shown potential for assisting in the treatment of neurodegenerative diseases such as Alzheimer’s disease (Ribeiro et al., 2022). Additionally, a dual responsive hydrogel based on poly N-isopropylacrylamide (PNIPAM) and polyacrylic acid (PAA) functionalized mesoporous silica nanoparticles (MSNs) has been shown to be effective for killing tumor cells while also promoting tissue regeneration (Chen et al., 2017). Further, studies have demonstrated that the incorporation of lipid nanoparticles into hyaluronic acid-functionalized hydrogels can help create an anti-inflammatory microenvironment and reduce immune response (Pavón et al., 2019). Currently, there is relatively limited research on the combination of hydrogels with nanomagnetic hyperthermia for neural injury repair. However, due to the potential benefits of hyperthermia, there may be significant applications in this area. We have found previous research demonstrating that a novel thermosensitive heparin-poloxamer (HP) hydrogel, delivering basic fibroblast growth factor (bFGF) and nerve growth factor (NGF), can be used for sciatic nerve compression injury in diabetic rats, promoting axonal and myelin sheath regeneration, and improving motor function recovery (Li et al., 2018). We believe that the combination of hydrogels with nanomagnetic hyperthermia has tremendous potential in neural injury repair and look forward to further progress in future studies (Pavón et al., 2019). Nanomaterials have shown promise in inhibiting the activity of inflammatory cells, which can help reduce inflammation, minimize immune reactions, and decrease immune rejection.

Table 3 provides a comprehensive overview of the growing body of research showcasing the effectiveness of using nanomaterials in combination with hydrogels for repairing neural injuries. These applications have been preliminarily validated in laboratory experiments, and some studies have progressed to animal and human trials. The combination of nanomaterials and hydrogels offers multiple advantages and has broad application prospects in neural tissue engineering. With further technological development, these materials are expected to play an even more significant role in the field of neural injury repair. It is excited to witness the potential of these materials and look forward to further advancements in this field.

**Table 3:** Representative research of varying hydrogels functionalized by nanoparticles and their biological performances in neural injury repairing.

Nanomaterials	Hydrogel	Types of study	Effects on biological performances	Ref.
MnO_2_	Hyaluronic acid hydrogel	*In vivo*	Remove active oxygen and increase the activity of mesenchymal stem cells, and enhance the efficient regeneration of spinal cord nerves	Li et al. (2019)
Polypyrrole	Collagen/hyaluronan hydrogel	*In vivo*	Antioxidation and conductivity enhance nerve regeneration and functional recovery	Wu et al. (2021)
Zinc-oxide	Chitosan hydrogel	*In vivo*	Improved mechanical strength and against infections	Joorabloo et al. (2022)
Polymeric gene	Hyaluronic acid hydrogel	*In vitro*	More mature neurons were engrafted to the host brain tissue	Li et al. (2016)
Mesoporous silica	Collagen hydrogel	*In vitro*	Neurite growth was improved	Lee et al. (2013)
Polypyrrole	N-isopropylacrylamide microgels	*In vivo*	Near-infrared-light responded delivery of glutamate	Li et al. (2015)
Iron oxide	Fibrin hydrogel	*In vitro*	Enhance the growth and differentiation neuronal precursor cells	Ziv-Polat et al. (2012)
Poly (lactic-co-glycolic acid)	Low/high-molecular-weight keratin hydrogel	*In vivo*	Improve the growth and differentiation of bone marrow mesenchymal stem cells	Gong et al. (2020)
Gold	Hyaluronic acid and pentenoate functionalized gelatin hydrogel	*In vitro*	Enhance the neural stem cells regeneration and reorganization	Kiyotake et al. (2022)
Silver	Polyacrylamide hydrogels	*In vivo*	Get electrical signals transmitted by neurons	Rinoldi et al. (2022)
Graphene oxide	Collagen hydrogel	*In vitro*	Promoting cell differentiation and inducing oriented cell growth	Santhosh et al. (2019)

Although nanomaterials hold great potential for the repair and regeneration of damaged tissues, their use in synthesizing new materials must consider potential immune responses and toxicity to tissues and organs, as well as issues of instability. Therefore, ensuring the biosafety and stability of nanomaterials is a crucial direction for future research in the field (Zheng et al., 2021).

The above paragraph describes different types of functionalized hydrogels, and the following materials have been specifically applied in cases of CNS or peripheral nervous system injuries. For instance, dopamine-modified chitosan hydrogels have been shown to improve cell survival, modulate immunity, and promote axonal regeneration (Liu et al., 2022). Additionally, the construction of injectable silk fibroin/dopamine hydrogels has been found to be helpful in promoting the repair of spinal cord injuries (Ye et al., 2021).

A novel conductive hydrogel made from gelatin methacrylate (GelMA), hyaluronic acid methacrylate (HAMA), and poly (3,4-ethylenedioxythiophene): sulfonated lignin (PEDOT: LS) has shown promise in repairing spinal cord injuries and promoting neuronal differentiation of neural stem cells (Gao et al., 2023). Additionally, bone marrow stem cell-derived exosomes (BMSC-exosomes) have been found to bind to conductive hydrogels, enhancing axonal growth and promoting tissue repair (Fan et al., 2022). The combination of a conductive hydrogel with chitosan to form a multifunctional double-layer hydrogel catheter has been shown to be effective for peripheral nerve injury repair (Deng et al., 2022). Finally, a rubber-like conductive hydrogel composed of gelatin, conductive polypyrrole, and tannic acid (referred to as GPT) has demonstrated the ability to promote peripheral nerve regeneration (Ye et al., 2021).

Injectable thermosensitive hydrogels containing immunoregulatory extracellular vesicles have been found to help alleviate inflammation and promote nerve regeneration in cases of spinal cord injury (Zhang et al., 2022). Additionally, the combination of exosomes derived from mouse neural stem cells with functional hydrogels based on poly (ε-caprolactone) has shown promise in promoting the survival and regeneration of spinal cord neurons, as well as reducing inflammation and glial scar formation (Xie et al., 2022). Another study found that the use of human mesenchymal stem cell-derived extracellular vesicles combined with chitosan-based functional hydrogels is effective in repairing peripheral nerve injuries in rats (Ju et al., 2023).

A composite hydrogel consisting of polyvinyl alcohol (PVA) and molybdenum sulfide/graphene oxide (MoS_2_/GO) nanomaterials has been shown to promote the differentiation of neural stem cells into neurons for use in spinal cord injury repair (Chen et al., 2022). Additionally, multifunctional biomimetic hydrogels based on graphene nanoparticles and sodium alginate have demonstrated the ability to simulate the microenvironment of nerve growth, reduce inflammatory factors, and contribute to the repair of peripheral nerve injuries (Ju et al., 2023).

## Conclusion

Biomedical materials for neural tissue engineering must possess not only excellent biocompatibility but also specific neural conduction functions that promote cell-to-cell signaling. Hydrogels, which are gel-like polymers containing water, represent the most water-like scaffold materials that closely resemble human soft tissue and are crosslinked by either physical or covalent bonds. However, conventional hydrogels exhibit low strength, weak biological activity, and a single function, limiting their ability to meet the complex demands of neural injury repair. Recent studies have demonstrated that functionalized hydrogels, created through combination with other functional materials, can improve biocompatibility and responsiveness to various stimuli by regulating their composition and structure. These functionalized hydrogels effectively mimic the *in vivo* neural extracellular matrix microenvironment, facilitating efficient repair, replication, or differentiation of neural cells and achieving high matching of the elastic modulus between the biomaterials and neural tissue. Despite their potential for aiding in the repair of nerve tissue after injury, functionalized hydrogels are still plagued by various deficiencies, such as concerns related to biosafety and stability. Additionally, their practical implementation in clinical applications remains an area of ongoing exploration. However, with continued advancements in technology, I am optimistic that these issues will be successfully resolved, and look forward to the further development and refinement of this exciting field.

## Author contributions

Conceptualization was made by JL and FZ. WZ and HT contributed equally to manuscript writing. HL and JW drafted the picture and summary the table. JC organized the material. All authors contributed to the article and approved the submitted version.

## Funding

This work was supported by the National Natural Science Foundation of China (no. 31800836) and Medical Health Science and Technology Project of Zhejiang Provincial Health Commission (Project number: 2019PY087).

## Conflict of interest

The authors declare that the research was conducted in the absence of any commercial or financial relationships that could be construed as a potential conflict of interest.

## Publisher’s note

All claims expressed in this article are solely those of the authors and do not necessarily represent those of their affiliated organizations, or those of the publisher, the editors and the reviewers. Any product that may be evaluated in this article, or claim that may be made by its manufacturer, is not guaranteed or endorsed by the publisher.
